# Quantitative Distributions of Product Ions and Reaction Times with a Binary Mixture of VOCs in Ambient Pressure Chemical Ionization

**DOI:** 10.1021/jasms.3c00189

**Published:** 2023-07-15

**Authors:** Elie Lattouf, Osmo Anttalainen, Oliver Hecht, Bert Ungethüm, Tapio Kotiaho, Hanna Hakulinen, Paula Vanninen, Gary Eiceman

**Affiliations:** † VERIFIN, Finnish Institute for Verification of the Chemical Weapons Convention, Department of Chemistry, University of Helsinki, FI-00014 Helsinki, Finland; ‡ AIRSENSE Analytics Gmbh, Hagenower Straße 73, 19061 Schwerin, Germany; § Drug Research Program and Division of Pharmaceutical Chemistry and Technology and Department of Chemistry, University of Helsinki, FI-00014 Helsinki, Finland; ∥ New Mexico State University, 1175 N Horseshoe Dr., Las Cruces, New Mexico 88003, United States

**Keywords:** atmospheric pressure ionization, hydrated proton, ionization selectivity, rate coefficient, vapor concentration, volatile organic compound

## Abstract

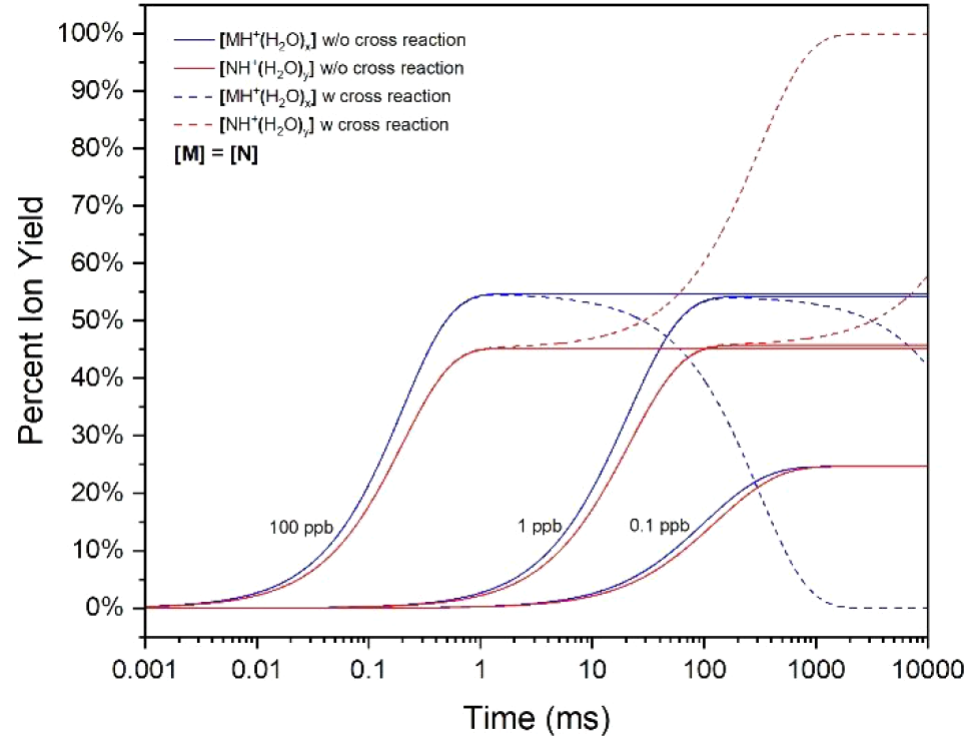

A model to quantitatively predict ion abundances from atmospheric pressure chemical ionization (APCI) between hydrated protons and a volatile organic compound (VOC) was extended to binary mixtures of VOCs. The model includes differences in vapor concentrations, rate coefficients, and reaction times and is enhanced with cross reactions between neutral vapors and protonated monomers. In this model, two specific VOCs were considered, a ketone, 6-methyl-5-hepten-2-one (M, and an amine, 2,6-di-*tert*-butyl-pyridine (N), with measured “conditional rate coefficients” (in cm^3^·s^–1^) of *k*_M_ = 1.11 × 10^–9^ and *k*_N_ = 9.17 × 10^–10^, respectively. The cross reaction of MH^+^(H_2_O)_*x*_ to NH^+^(H_2_O)_*y*_ was measured as *k*_cr_ = 1.31 × 10^–12^ at 60 °C. Cross reactions showed an impact on ion abundances at *t* > 30 ms for equal vapor concentrations of 100 ppb for M and N. In contrast, this impact was negligible for vapor concentrations of 1 ppb and did not exceed 5% change in product ion abundance up to 1000 ms reaction times. The model was validated with laboratory measurements to within ∼10% using an ion mobility spectrometer and effective reaction time obtained from computational fitting of experimental findings. This was necessitated by complex flow patterns in the ion source volume and was determined as ∼10.5 ms. The model has interpretative and predictive value for quantitative analysis of responses with ambient pressure ion sources for mass spectrometry and ion mobility spectrometry.

## Introduction

Chemical measurements in mass spectrometry (MS) with ion sources at ambient pressure can be attributed to the introduction of atmospheric pressure ionization (API) MS in 1973.^1^ Applications with pharmaceutical and environmental samples were encouraged by low detection limits, high selectivity of response controlled ion–molecule reactions (i.e., gas phase basicity), and technology simplicity with corona discharges or β-emitters.^2,3^ Today, the principles of API MS can be associated with a large number of ion sources at ambient pressure^4,5^ and ionization may be described as displacement reactions as shown in eqs 1 and 2:
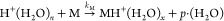
1
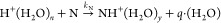
2where sample neutrals (M or N) react with hydrated protons, forming excited intermediates, e.g., (MH^+^(H_2_O)_*x*_)*, which are stabilized by the loss of water adducts to product ions via collisions with neutral gas molecules, here MH^+^(H_2_O)_*x*_ and NH^+^(H_2_O)_*y*_. Quantitative response in API-MS was shown to be roughly proportional to gas phase basicity (GB) below 790 kJ/mol and constant above this value.^2^ Exceptions to a linear correlation between response and GB were corrected by increasing the temperature of the source.^3^

Reactions are relatively fast with collision frequencies of roughly 7 × 10^9^ s^–1^ for hydrated protons and neutrals following second order kinetics as described recently with a general model for ion abundances, reaction times, rate coefficients, and vapor concentrations.^6^ Findings from this computational model demonstrated that reactions reach completion in tens to hundreds of milliseconds at parts per billion (ppb) vapor concentrations, and these are significantly longer than sample residence times in commercial ion sources for MS and ion mobility spectrometry (IMS). Ionization of sample neutrals M and N to product ions follow second order rate equations (eqs 3 and 4):
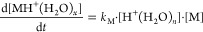
3
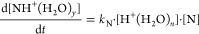
4with characteristic rate coefficients *k*_M_ and *k*_N_ at fixed values of moisture and temperature. Rate coefficients have been reported for proton transfer reaction mass spectrometry,^7^ flowing afterglow,^8−10^ or selected ion flow tube mass spectrometry^11,12^ and computed for H^+^(H_2_O) with a large number of VOCs;^13^ rate coefficients at ambient pressure are largely unreported apart from a few values.^14,15^ The unavailability of relevant rate coefficients is noted here as an obstacle to building an interpretative and predictive model for API-MS for single constituents or mixtures.

Only few quantitative studies for reactions in eqs 1 and 2 with mixtures have been described using API MS^16^ and IMS,^17^ and none can be extended generally to other mixtures and measurements where gas phase ionization can be described as competitive proton exchange and measured ion abundances do not necessarily reflect the molar composition of a sample. This process of competitive proton exchange among constituents in a sample can contribute to what is known as the matrix effect^18^ or suppression of ionization,^19,20^ and such processes commonly occur with electrospray ionization in solution rather than in the gas phase.^21^ A quantitative description for gas phase competitive ionization with mixtures with API MS was found for a binary mixture of organophosphorus compounds.^22^ The predictive model based on differential equations for ion formation and ion losses favorably matched ion abundances for dimethyl methyl phosphonate in the presence of increasing levels of di-isopropyl methyl phosphonate.

Competitive proton exchanges in only the gas phase and the consequences for chemical measurements have been described significantly with IMS methods.^17,23−25^ Efforts to provide practical solutions have included continuous flow standard addition^26^ and the use of alternate reagent gases or dopants in IMS.^27,28^ Chemical reagents added into sources have been used also with MS for atmospheric pressure photoionization (APPI)^29^ and in the development of a multireagent ion source.^30^

Critically, the topic of response with mixtures has not been approached quantitatively by using a computational model that has predictive or interpretative capabilities. A foundation provided for a single constituent has been extended here for a binary mixture (2,6-di-*tert*-butyl-pyridine and 6-methyl-5-hepten-2-one) and validated experimentally. The study includes measurements of rate coefficients for individual substances and a favorable cross reaction. Objectives through computational modeling are

1. Determine relative ion abundances and times of reactions for binary mixtures with differences in rate coefficients and vapor concentrations.
2. Explore the impact of reactant ion density H^+^(H_2_O)_*n*_ as the limiting reagent in eqs 1 and 2.
3. Validate *in silico* modeling with experimental results for binary mixtures, and
4. Introduce the concept of a conditional rate coefficients, i.e., *k* specific to a set of experimental conditions, since moisture and temperature influences the distribution and reactivity of hydrated protons in air at ambient pressure.

## Experimental Section

### Instrumentation

#### Ion Mobility Spectrometer to Determine Rate Coefficients

An ion mobility spectrometer (model GDA-P, AirSense Analytics, Schwerin, Germany) was modified with a unidirectional flow of drift gas from the detector to the reaction region. A membrane pump (model NMP-015.1.2, KNF, Freiburg im Breisgau, Germany) was used to establish a drift gas flow of 241 mL·min^–1^ of air in a recirculated flow design with flow vented through the ion source (Figure S1). Flows were purified by using a molecular sieve filter. The drift tube voltage was varied between 2 and 2.5 kV to control reaction times as described by Valadbeigi et al.^15^ The gas temperature was maintained at 60 °C. Spectra were acquired by using Winmuster (Airsense Analytics). The ion source was ^63^Ni (95 MBq) and the ion shutter was a Bradbury-Nielson design. This same drift tube was used to determine rate coefficients of a cross reaction (eq 5) and was modified to introduce 6-methyl-5-hepten-2-one [M] into the reaction region and 2,6-di-*tert*-butyl-pyridine [N] into the drift region (Figure S2).
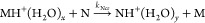
5A rate coefficient for the backward cross reaction (eq 6) was not determined in this study because such reactions are negligible:
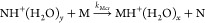
6

#### Ion Mobility Spectrometer to Validate the Computational Model

An ion mobility spectrometer was built in-house (Figure S3) from a general structure and materials already described in detail and with a few modifications.^31^ The ion source was a corona discharge and data acquisition was made using an 18-bit PCI-6281 DAQ interface card (National Instruments Corp., Austin, TX, USA) and a LabView program developed in-house and labeled Linear 2018 version 2.0. Data were acquired at 166.7 kHz for 28 ms spectra with a temporal resolution of 6 μs and a spectral averaging of 20 ms. The pulse width used for opening and closing the ion shutter was set to ∼120 μs. Note that this instrument has two ion shutters with the second one continuously kept open (i.e., inactive) during the measurements. The drift tube was combined with a Model 6890 gas chromatograph (Agilent Corp., Avondale, PA, USA) equipped with a 15 m RTX-5 capillary column with a nitrogen carrier gas. The drift gas (air) was purified using activated carbon and molecular sieves with a flow of 380 mL.min^–1^ and at source vent, the drift gas temperature was raised to 60 °C by heating the flange to a temperature of 120 °C.

### Chemicals and Solvents

One compound, 2,6-di-*tert*-butyl-pyridine, was obtained from Sigma-Aldrich Company (Steinheim, Germany) with ≥97% purity. A second compound, 6-methyl-5-hepten-2-one, was obtained at 99% purity from Sigma-Aldrich or from BLDpharm (Pudong, China). The solvent methylene chloride, which was used as a standard, was purchased from Sigma-Aldrich with a stated purity being ≥99.5%.

### Procedures

#### Experimental Determination of Conditional Rate Coefficients for Individual Compounds Using Ion Mobility Spectrometry

A method to determine rate coefficients for the formation of proton bound dimers^32^ was used here for the formation of protonated monomers. Three microliter volumes of either 6-methyl-5-hepten-2-one or 2,6-di-*tert*-butyl-pyridine were vaporized singly into gastight sealed bags (Studio Cook roasting bags, Bilthoven, Netherlands) filled with purified air. Headspace volumes of 76 mL for 2,6-di-*tert*-butyl-pyridine and 50 mL for 6-methyl-5-hepten-2-one were diluted into fresh bags. Vapor concentrations were determined using a piD-TECH eVx silver analyzer (AMETEK MOCON, Ringsted, Denmark) as 1.67 ± 0.05 and 1.35 ± 0.04 ppm, respectively. A vapor sample was delivered using a 10 mL Fortuna Optima gastight syringe (Poulten & Graf GmbH, Wertheim, Germany) and a syringe pump (Cole-Parmer 78–8100C, Vernon Hills, IL, USA) into the drift tube at 0.1–0.8 mL·min^–1^ providing final concentrations in the drift gas were 5.55 ± 0.17 and 4.50 ± 0.14 ppb, respectively. After 20 min, a steady state was observed experimentally, and a rate coefficient (*k*) is determined from the baseline slope (*m*) between the reactant ion and product ion (e.g., 6-methyl-5-hepten-2-one [M]) of the natural logarithm of ion intensity in the ion mobility spectrum.
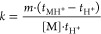
7where [M] is the vapor concentration and *t*_MH^+^_ and *t*_H^+^_ are drift times for respective ion peaks. Rate coefficients were obtained at various electric field strengths (from 332 to 420 V·cm^–1^) at a fixed vapor concentration.

A rate coefficient for the cross reaction between protonated ketone and an amine neutral (eq 5) was determined using a flow of ketone in the ionization region and an amine into the drift region (Figure S2). The same procedure was utilized for sample preparation, and final vapor concentrations were 2 and 2.3 ppm for 6-methyl-5-hepten-2-one and 2,6-di-*tert*-butyl-pyridine, respectively.

#### Experimental Validation of Model by Gas Chromatography-Ion Mobility Spectrometry

Standard solutions of 6-methyl-5-hepten-2-one in methylene chloride providing in-source vapor concentrations of 3.5–15 ppb were analyzed by GC-IMS to establish response curves. Then, a constant flow of headspace vapors from neat 6-methyl-5-hepten-2-one vapor was delivered into the IMS drift tube. The vapor concentration was determined to be 1.22 ppb from the response curves and standard solutions for 2,6-di-*tert*-butyl-pyridine in methylene chloride were analyzed using this GC-IMS. The column temperature was ramped from 70 to 120 °C at 10 °C·min^–1^. The injection port temperature was 150 °C with a splitless injection time of 0.5 min. Sample volumes were 10 μL, and standards were prepared so that a residual intensity of the reactant ions was observed, even at the maximum for the chromatographic elution profile.

Measurements were analyzed for response peak intensity from IMS data and plotted as a function of experiment time for hydrated protons, 6-methyl-5-hepten-2-one, and 2,6-di-*tert*-butyl-pyridine. The near-Gaussian elution profile from the GC^33^ was treated as a histogram of response maxima. These maxima were used to calculate vapor concentrations for 2,6-di-*tert*-butyl-pyridine delivered into the reaction region of the IMS, ranging between 0.05 and 3.16 ppb.

These vapor concentrations from the experimental studies were introduced into the *in silico* model where 6-methyl-5-hepten-2-one [M] was set to 1.22 ppb and [N] was set to values of concentrations throughout the elution profile of 2,6-di-*tert*-butyl-pyridine.

#### Computational Studies

##### MatLab

Initial conditions were loaded in defined variables, and the system of differential equations (eqs 8–10; or eqs 11–14 when cross reactions are included) was introduced into MatLab R2021b from MathWorks and solved using ode45 solver. Both relative and absolute tolerance values were set as low as 2.5 × 10^–14^ and only “NonNegative” solutions were allowed. This drives the solver toward small time steps to avoid depleting analyte concentrations in a single step; thus, divergent solutions and negative vapor concentrations are avoided. Results were organized in matrices and exported in ASCII or text files. The matrices were imported into OriginPro 2021b SR2 9.8.5.212 from OriginLab corporation for plotting.

### Details of the Model

The model for APCI reactions is based on binary reaction kinetics between hydrated protons and analytes (M, the ketone and N, the amine) in air at or near ambient pressure with certain assumptions and limitations:

1. Reactant ions, H^+^(H_2_O)_*n*_ are extracted from an ion source and mixed with sample vapors (M) and (N). The system is closed without sample loss or ion loss by ventilation or neutralization on surfaces. Recombination losses are also neglected since there are no negative species in the reaction volume.
2. Charge density for hydrated protons is set at an initial value of 1 × 10^10^ cm^–3^ and decreases without any further addition of charge during the reaction time.^34^ This means that the rate of production of reagent ions is assumed to be much smaller than the rate of consumption.
3. Losses from diffusion have been omitted in the model due to the small reaction times and low product ion yield.
4. Moisture and temperature are “fixed” so the water cluster size *n*, in H^+^(H_2_O)_*n*_, is considered as the average *n* = 3. Thus, a single “weighted” rate coefficient, a conditional rate coefficient, is reported for an individual compound from the overall reaction with ∼3 hydrated protons clusters (*n* = 2–4).
5. Ion density for hydrated protons is changed only through reactions and extraction from the reaction volume; there is no change by dilution with gas flows. Changes in densities for neutrals and hydrated protons are given in eq 8 through eq 10.
6. The product ion lifetime exceeds the duration of experiments; thus, backward reactions are ignored. That is *k*_reverse_ ≪ *k*_forward_ in eqs 1 and 2.
7. Proton bound dimers and heterogeneous dimers are not significantly formed by compounds of interest in this study and are not included in the model. Thus, higher order cluster ions such as proton bound trimers and tetramers are also not considered.
8. For the studies with cross reactions, forward reaction (eq 5) was considered solely and backward cross reaction (eq 6) was considered as inconsequential (*vide infra*).
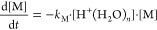
8
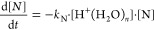
9
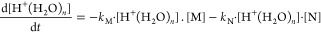
10

Four in-silico experiments were used in this study and included:

1. **Influence of Vapor Concentration for Binary Mixture with Empirical*k***. Vapor concentrations of M and N were matched at two levels 0.1 and 1 ppb for hydrated protons in excess or as limiting reagent, respectively. Since the reactivity of hydrated protons is governed by hydration level, rates obtained experimentally, at fixed temperature and moisture, are termed “conditional rate coefficients” and used in further calculations in this model.
2. **Effect of Relative Neutral Densities of Sample**. Hydrated protons levels and product ion yield were explored for reaction times up to 1000 ms using the same conditional rate coefficients for the mixture of M and N. Vapor levels for [N] were fixed at 0.1 and 1 ppb, whereas the vapor concentration of M was varied from 0.01 to 100 ppb.
3. **Contributions to Ion Abundances from Cross Reactions** Three equimolar levels of M and N were modeled with the addition of the forward cross reaction shown in eq 5. The rate coefficient for the reverse cross reaction (eq 6) was unmeasurable, possibly from steric hindrance by the two *tert*-butyl groups of 2,6-di-*tert*-butyl-pyridine. Thus, there was no close approach of ketone to the protonated ring nitrogen. Rate equations shown previously without the cross reaction become eqs 11–^14^.
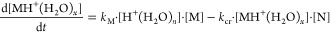
11
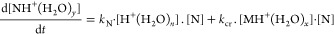
12
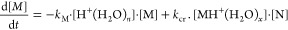
13
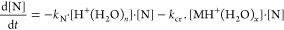
14
4. **Validation of Model at Specific Conditions**. Quantitative response in the ion source of a mobility spectrometer was obtained with constant vapor concentration of M at 1.22 ppb and those for [N] were distributed over the elution profile from chromatographic retention of N.
5. **A General Model for Binary Vapor Mixtures**. In general studies with the model, values for [M] and [N] were both at 10 ppb. Other terms were [H^+^(H_2_O)_*n*_], at 406 part per trillion, or ppt (1 × 10^10^ cm^–3^); and *k*_N_, 1 × 10^–10^ cm^–3^. The value for *k*_M_ was varied from 1 to 50 × 10^–10^ cm^–3^ with reaction times from 0.1 to 1000 ms.

## Results and Discussion

### Influence of Vapor Concentration for Binary Mixture with Measured Rate Coefficients

#### Conditional Rate Coefficients in Air at Ambient Pressure

**Figure 1:**
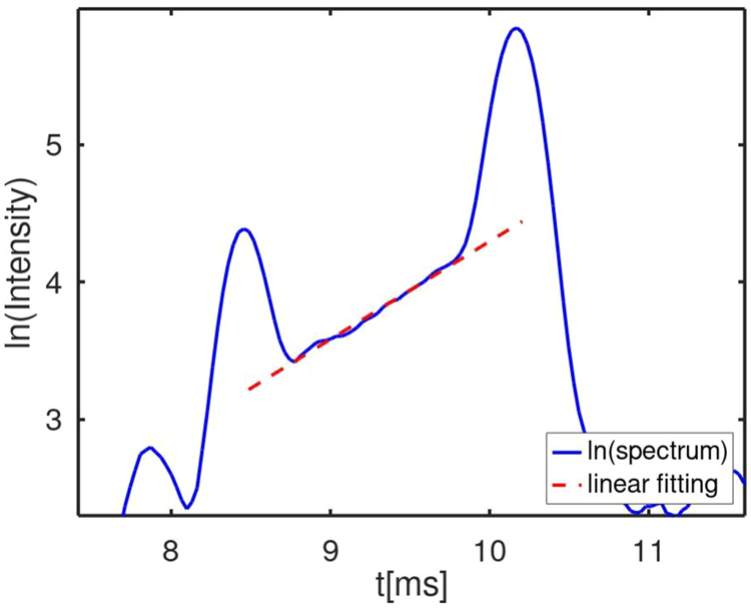
Ion mobility spectrum with 6-methyl-5-hepten-2-one introduced in the drift gas flow. Reactant ions appear at 8.5 ms, and protonated monomer at 10 ms; the peak at 7.9 ms is that of the protonated monomer of ammonia.

The natural logarithm of ion intensity in an ion mobility spectrum is shown in Figure 1. The rate coefficient *k*_M_ (eq 1) for 6-methyl-5-hepten-2-one was obtained following the method first shown by Tabrizchi and co-workers.^15,32^ The peak at ∼8.5 ms is that of hydrated protons, and the one at 10.18 ms is that of (MH^+^(H_2_O)_*x*_) formed in the reaction region. The baseline rise is due to the formation of the protonated monomer as the vapor moves counterflow through the drift region.

Reactant ions which enter the drift region will react with the neutral sample in the drift region on their way to the detector, forming additional product ions. These product ions will reach the detector at drift times between those of the reactant ions and already existing product ions due to the shorter drift distance from the point of their formation. Thus, an increase of the detected ion current between the reactant ion and product ion peak is detected. Due to the consumption of reactant ions as a consequence of this proton transfer reaction in the drift region, the probability of product ion formation decreases in the drift direction. This causes a positive slope for the ion current intensity between the reactant ion and product ion peak.

A similar profile for *k*_N_ (eq 2) was obtained for 2,6-di-*tert*-butyl-pyridine. Conditional rate coefficients were extracted from the baseline slopes as described above and were 1.11 × 10^–9^ cm^3^·s^–1^ for the 6-methyl-5-hepten-2-one (*k*_M_ for compound M) and 9.17 × 10^–10^ cm^3^·s^–1^ for 2,6-di-*tert*-butyl-pyridine (*k*_N_ for compound N). While the reactivity for 2,6-di-*tert*-butyl-pyridine (PA 982.9 kJ/mol^35^) should be more than that of 6-methyl-5-hepten-2-one (PA calculated as 875 kJ/mol by DFT from Orca Software), the two *tert*-butyl groups are understood to sterically hinder reactions with proton clusters and result in a lower rate coefficient than that for the unhindered protonation site (C=O) on the ketone. These conditional rate coefficients, which are influenced by levels of hydration, are comparable to those reported with MS for specific hydrated protons^8−10,36,37^ and such dependences are also shown from studies in IMS.^38^

**Figure 2:**
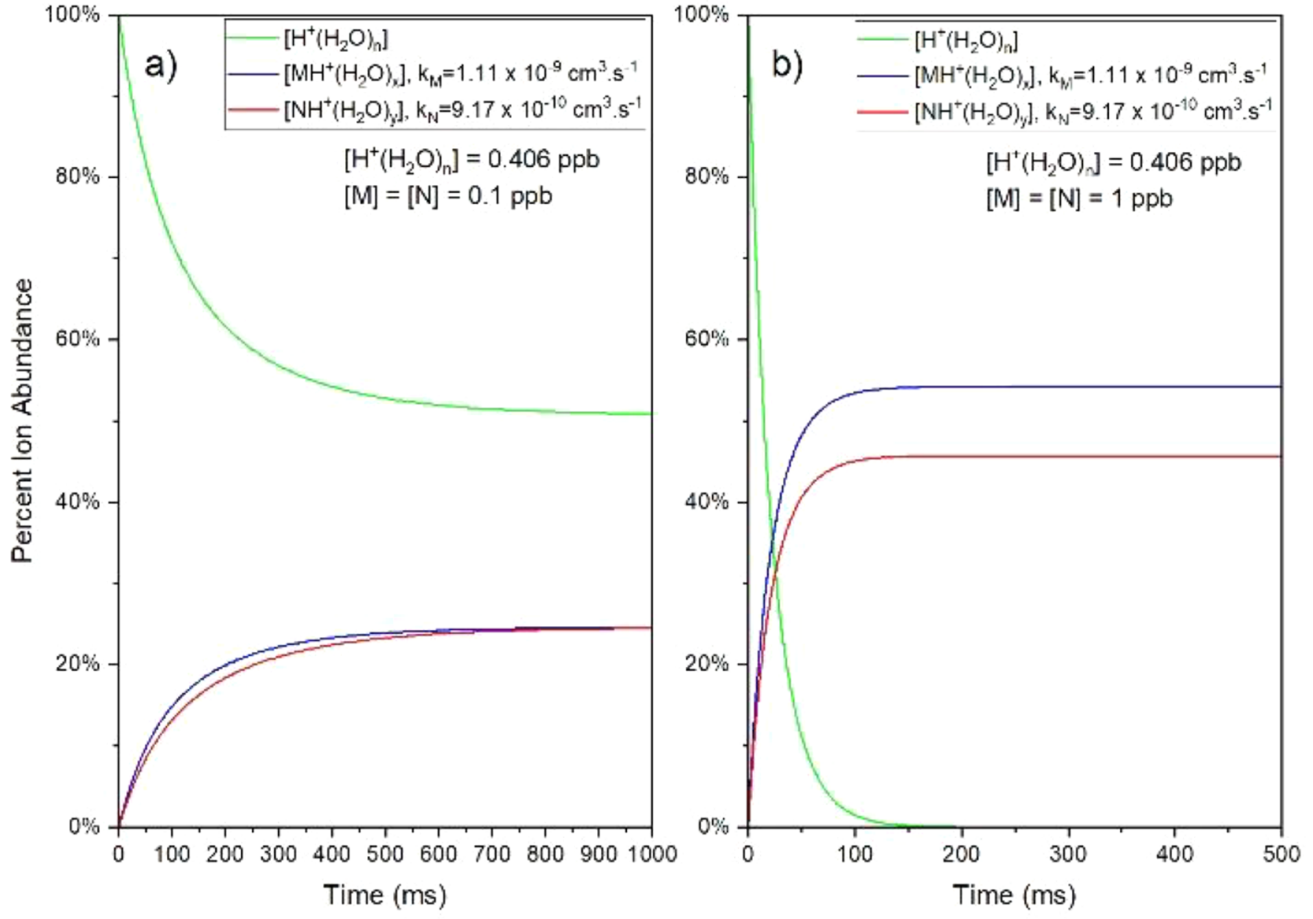
Percent of ion abundance with reaction time using empirical conditional rate coefficients for equal vapor concentrations of M and N. (a) Left plot: total vapor concentration 0.2 ppb. (b) Right plot: total vapor concentration 2 ppb.

Differences in product ion abundances were explored with hydrated protons as excess *or limiting* reagent and where initial vapor concentrations were [M]_i_ = [N]_i_ = 0.1 ppb or [M]_i_ = [N]_i_ = 1 ppb. Plots of percent ion abundances vs time are shown in Figure 2a and b, respectively, for these two initial conditions.

Ion abundances reflect pseudo first order kinetics at low reaction times (50 ms and below at left and 5 ms or below at right); above these times, the reaction shows complete second order behavior until reactant ions are fully consumed. Ion abundances with 0.2 ppb total vapor concentrations show completion above 800 ms (at left) and between 150 to 200 ms with 2 ppb total vapor concentrations (at right). Charge is conserved in total ion abundance without recombination in a unipolar ion environment.

Steady state is reached in both instances due to the limiting reagent. At the left, with a total vapor concentration of 0.2 ppb, the vapors are the limiting reagent, and a steady state is reached without complete depletion of reactant ions. At the right, with a total vapor concentration of 2 ppb, the hydrated protons are the limiting reagent with a density of only 406 ppt and depletion of reactant ion occurs. In both instances, the ratio of product ion abundances matches the ratio of the rate coefficients *k*_M_/*k*_N_ ≈ 1.21 at t = 0 ms. This ratio drops ∼10% in favor of the amine ion (N) at 2 ppb with reactant ion depletion; in contrast, the ratio approaches 1 asymptotically at ∼1000 ms without reactant ion consumption (Figure S4).

Although modeling was restricted to binary mixtures with low-ppb levels of equal vapor densities, the model is applicable to a large range of vapor concentrations where ion abundances and reaction times are dependent on relative vapor concentrations of constituents.

### Effect of Relative Neutral Densities of Sample

**Figure 3:**
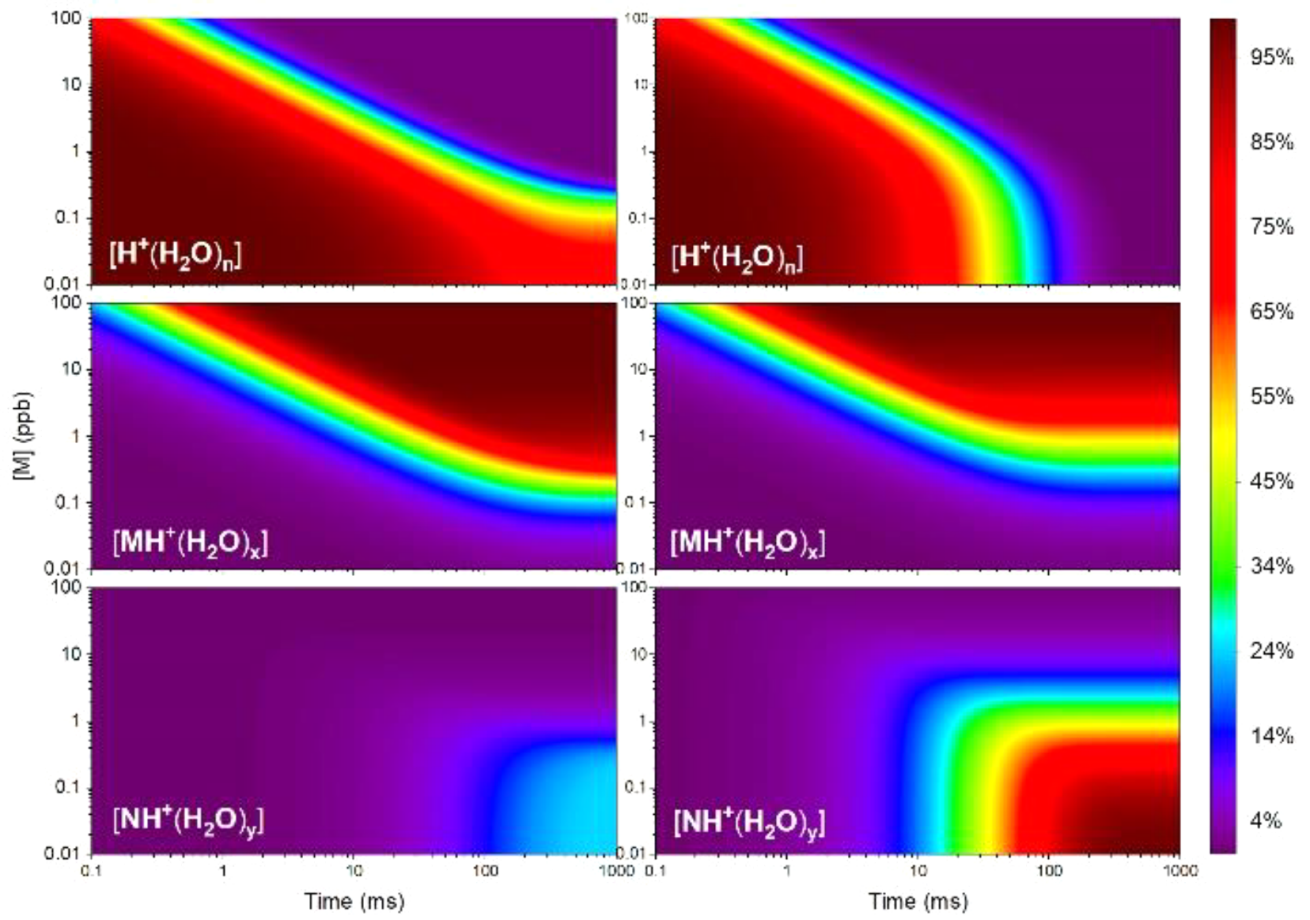
Influence of vapor concentration of M on percent ion yield of the ketone M and the amine N for empirical values of rate coefficients, with fixed values of amine density [N]. (Left [N] = 0.1 ppb, right [N] = 1 ppb.)

Plots are shown in Figure 3 for ion abundances of the hydrated protons, [MH^+^(H_2_O)_*x*_], and [NH^+^(H_2_O)_*y*_] over a range of vapor concentrations of M, with [N] = 0.1 ppb for the left plots and [N] = 1 ppb at right. In the plot at the top left, hydrated protons are always in excess when [M] is below 0.306 ppb.

Ion abundances for MH^+^(H_2_O)_*x*_ are observed (∼10% or greater yield|) throughout the reaction times as long as vapor concentrations are greater than 0.08 ppb. Reaction times to form MH^+^(H_2_O)_*x*_ decrease with increases in vapor concentrations and reach times of ∼0.2 ms with [M] = 100 ppb. In contrast, NH^+^(H_2_O)_*y*_ is largely not observed above a few percent anywhere in the plot except when [M] is below 0.83 ppb and reaction times are greater than 80 ms. Maximum percent yield for NH^+^(H_2_O)_*y*_ reaches a plateau of ∼24% at times > 400 ms (left bottom frame in Figure 3). This percent ion yield corresponds to full conversion of neutral analyte N into product ion NH^+^(H_2_O)_*y*_.

These patterns are similar when the hydrated proton is the limiting reagent (at right in Figure 3) with a few significant differences compared to Figure 3 (left frame). Reactant ions are completely depleted in the ion source region after about 400 ms, even when [M] is as low as 0.01 ppb. At reaction times greater than 8 ms, ion abundances for M and N are the same when [M] = 0.826 ppb. At vapor concentrations for M below this, the principal ion is NH^+^(H_2_O)_*y*_ reaching 100% yield with [M] below 0.1 ppb while MH^+^(H_2_O)_*x*_ is the principal ion at all other vapor concentrations.

The abundances of individual product ions in absolute and relative intensities are governed by eqs 3 and 4; consequently, the distribution of protons is going to follow (rate coefficient) × (vapor concentration). Moreover, the relative abundances follow as a ratio of *k*_M_[M]/*k*_N_[N] and the pattern of distribution of protons between MH^+^(H_2_O)_*x*_ and NH^+^(H_2_O)_*y*_ is seen according to this ratio in both the left and right contour plots. Differences arise through the vapor concentration of N. At 1 ppb for [N] at the right, competitive exchange of protons in favor of [N] arises only when [M] < (*k*_N_ /*k*_M_) × [N], or [M] < 0.826 ppb, and this occurs at times above 4 ms. For any other vapor concentration of M above this value, N does not compete significantly for protons at any reaction time.

**Figure 4:**
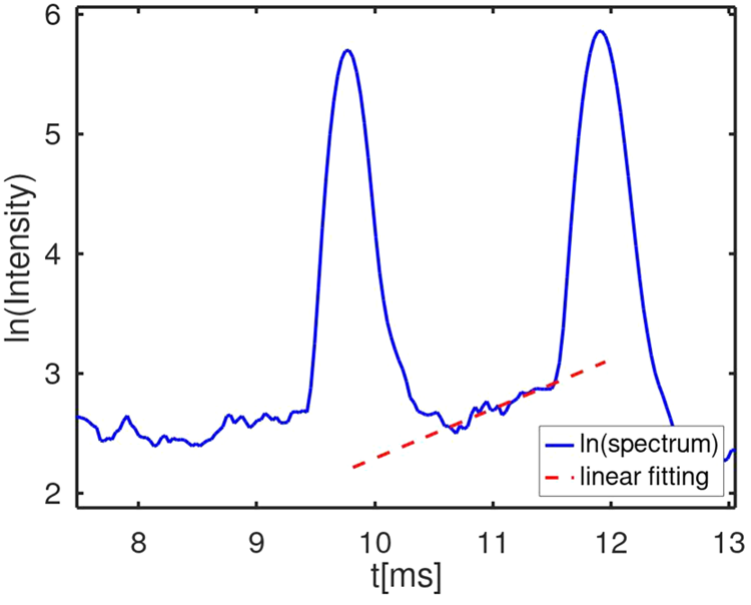
Mobility spectrum for the determination of *k*_cr_ with 6-methyl-5-hepten-2-one (peak at ∼9.8 ms) as reagent ion and 2,6-di*tert*-butyl-pyridine vapor. The latter appears at ∼11.9 ms.

Although rate coefficients in API systems may differ by 1 to 2 orders of magnitude, vapor concentrations of constituents in mixtures in practical chemical analyses may differ by 10^5^ or more. *Consequently*, vapor concentration is the primary practical variable in API MS or IMS methods for mixtures. While the results in Figures 2–4 are simplified without cross reactions or proton transfer between product ions, any effort to experimentally validate the model will necessitate rate coefficients for cross reactions.

### Experimental Validation of Model for Binary Mixtures

#### Experimental Determination of Cross Reaction Rate Coefficient

**Figure 5:**
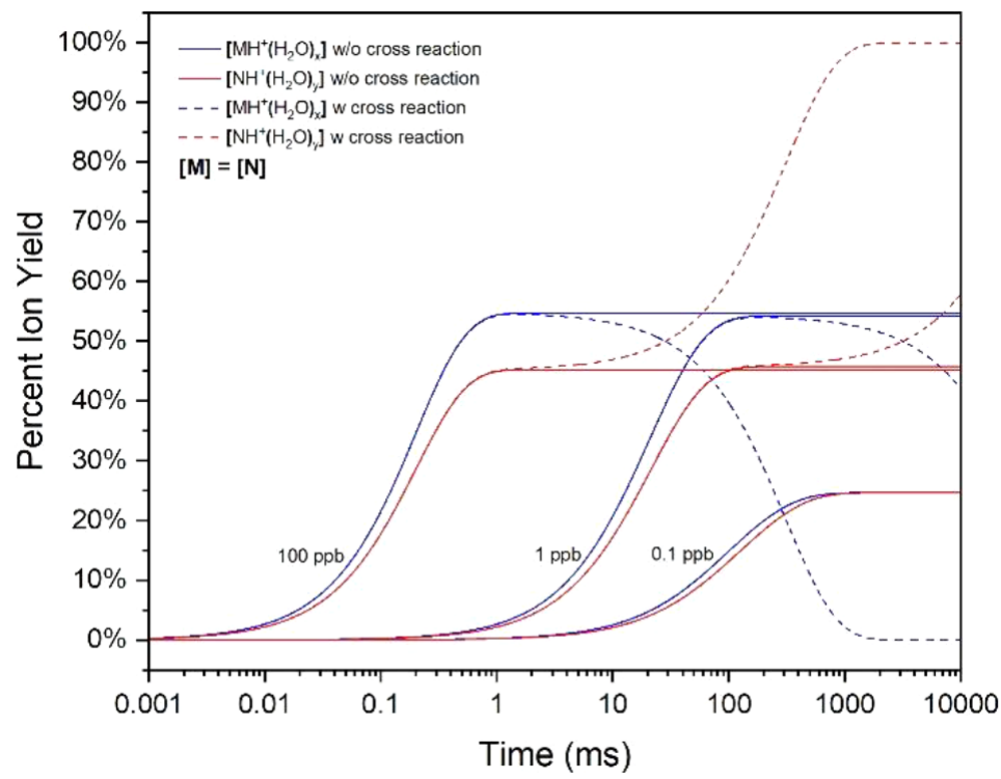
Percent ion yield with binary mixture with (solid lines) and without (dashed lines) cross reaction at 0.1, 1, and 100 ppb equimolar concentration levels.

A natural logarithm of ion intensity in a mobility spectrum (Figure 4) is shown for protonated 6-methyl-5-hepten-2-one and protonated 2,6-di-*tert*-butyl-pyridine where 6-methyl-5-hepten-2-one was added to the ion source, so all reactant ions were consumed, and 2,6-di-*tert*-butyl-pyridine was added to the drift gas with a counterflow (Figure S2). The peaks have widths and symmetry characteristic of mobility measurements, and the elevated baseline between the peaks (inset) is suitable to determine a rate coefficient for the reaction in eq 5. The baseline slope in the natural logarithm of ion intensity in the spectrum provides a value for *k*_Mcr_ = *k*_cr_ which was 1.31 × 10^–12^ cm^3^·s^–1^ at 60 °C. This rate coefficient is consistent with the displacement of a ketone with an amine arising from a higher enthalpy of association of the proton with a nitrogen base than with an oxygen base.^39^ The magnitude of *k*_cr_ is more than 2 orders of magnitude smaller than reactions of either substance with a hydrated proton (*k*_M_ = 1.11 × 10^–9^ cm^3^·s^–1^ and *k*_N_ = 9.17 × 10^–10^ cm^3^·s^–1^); nonetheless, the impacts of the cross reaction should be included and explored. The influence from cross reactions with binary mixtures is shown in plots of ion yields with reaction time, with and without cross reactions, for individual vapor concentrations of 0.1, 1, and 10 ppb (Figure 5). At 0.1 ppb, plots for MH^+^(H_2_O)_*x*_ and NH^+^(H_2_O)_*y*_ are indistinguishable from yield, regardless of corrections for the cross reaction. At 1 ppb, decreases in ion yield for MH^+^(H_2_O)_*x*_ occur above 100 ms, while increases occur proportionally for [NH^+^(H_2_O)_*y*_] with cross reaction. The dominant ion when reaction times exceed 3000 ms is NH^+^(H_2_O)_*y*_ when cross reactions are included (dashed lines). At 100 ppb, also decreases in ion yield for MH^+^(H_2_O)_*x*_ occur above 1 ms with cross reaction while increases occur proportionally for [NH^+^(H_2_O)_*y*_] with inversion of dominance of product ions at ∼30 ms. A plateau is reached above 1000 ms for NH^+^(H_2_O)_*y*_ and MH^+^(H_2_O)_*x*_ to drop to zero.

At low vapor concentrations of 0.1 ppb, the hydrated protons are in excess, neutral [N] approaches zero, and cross reactions cease. In contrast, at increased vapor concentrations of 1 or 100 ppb, [N] is in excess and increases in collision frequencies occur where N displaces M on MH^+^(H_2_O)_*x*_. Mathematically, these cross reactions continue over time as long as both reagents of eq 5 are available. The effect of diminished cross reaction is seen at 0.1 ppb with vapors being the limiting reagents, and where a lack of N neutrals stops the reaction in eq 5 from occurring. This is also seen at 100 ppb, above 1000 ms, where MH^+^(H_2_O)_*x*_ becomes the limiting reagent of the reaction in eq 5.

While vapor concentration was shown to control the formation of higher clusters such as proton bound dimers,^6^ this second pathway (cross reaction) also occurs proportionally. Such cross reactions will certainly be present with substances that do form higher clusters, and we can anticipate heterogeneous proton bound dimers (i.e., MNH^+^) along with M_2_H^+^ and N_2_H^+^. This has already been observed by Ewing et al.^40^ for vapor mixtures of small alcohols, although no computational models for interpretation or prediction were provided.

#### Laboratory Validation of Model for Binary Mixtures

**Figure 6:**
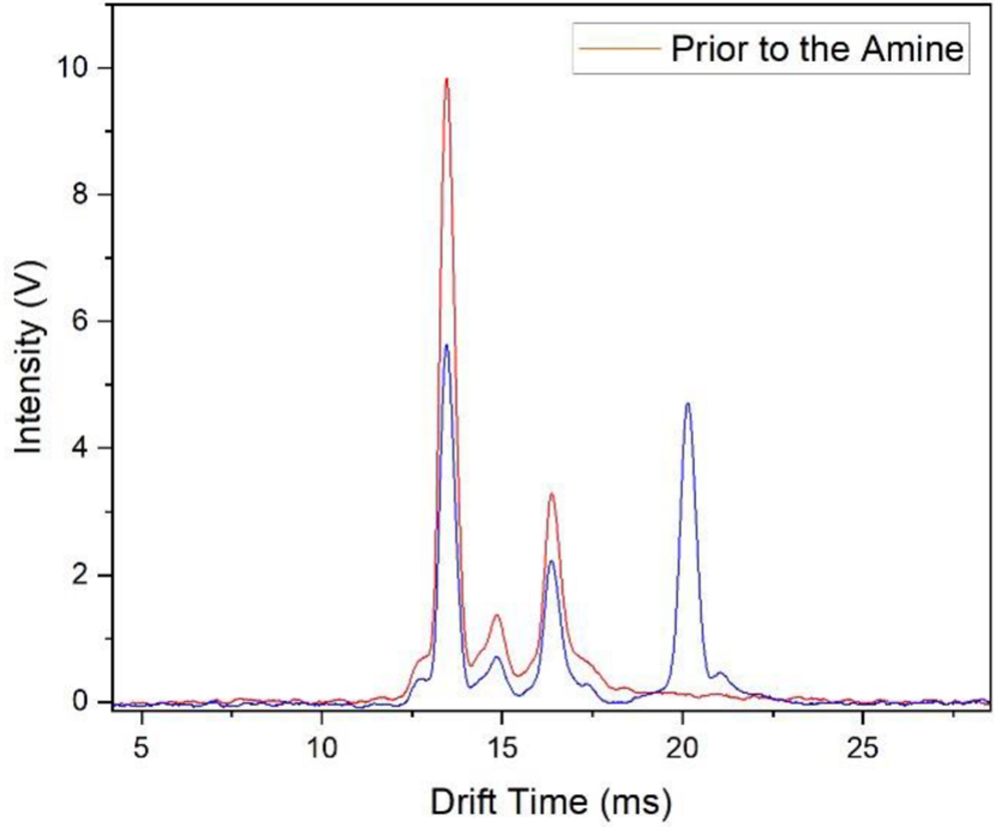
Ion mobility spectra for a single compound, 6-methyl-5-hepten-2-one alone (red), and with 2,6-di*tert*-butyl-pyridine (blue).

Mobility spectra are shown in Figure 6 with a constant vapor concentration of M only (red) and for a binary mixture when N is introduced with gas chromatographic effluent (blue). The spectrum for the binary mixture (blue) is shown at the elution profile maximum where the vapor concentration of N is the highest. In the spectrum for M only, the reactant ion peak for H^+^(H_2_O)_*n*_ is seen at 13.46 ms, and the peak for MH^+^(H_2_O)_*x*_, formed through eq 1, is seen at 16.36 ms. A contaminant in the ketone is observed at 14.84 ms and was identified by using accurate mass spectrometry as 2,7-octanedione (Figure S5). The abundance of the contaminant was 2% by mass and was ∼25% of the ketone by response with GC MS. APCI MS measurements of headspace from the ketone showed an intense protonated monomer for the 2,7-octanedione at *m*/*z* = 143 Da (Figure S6). Combining percent mass with APCI response, which is directly proportional to k x [ ], leads to an estimated rate coefficient for ionization of the 2,7-octanedione of *k*_cont_ = 12.5*k*_M_ = 1.39 × 10^–8^ cm^3^·s^–1^, explaining the highly reactive aspect of the contaminant.

**Figure 7:**
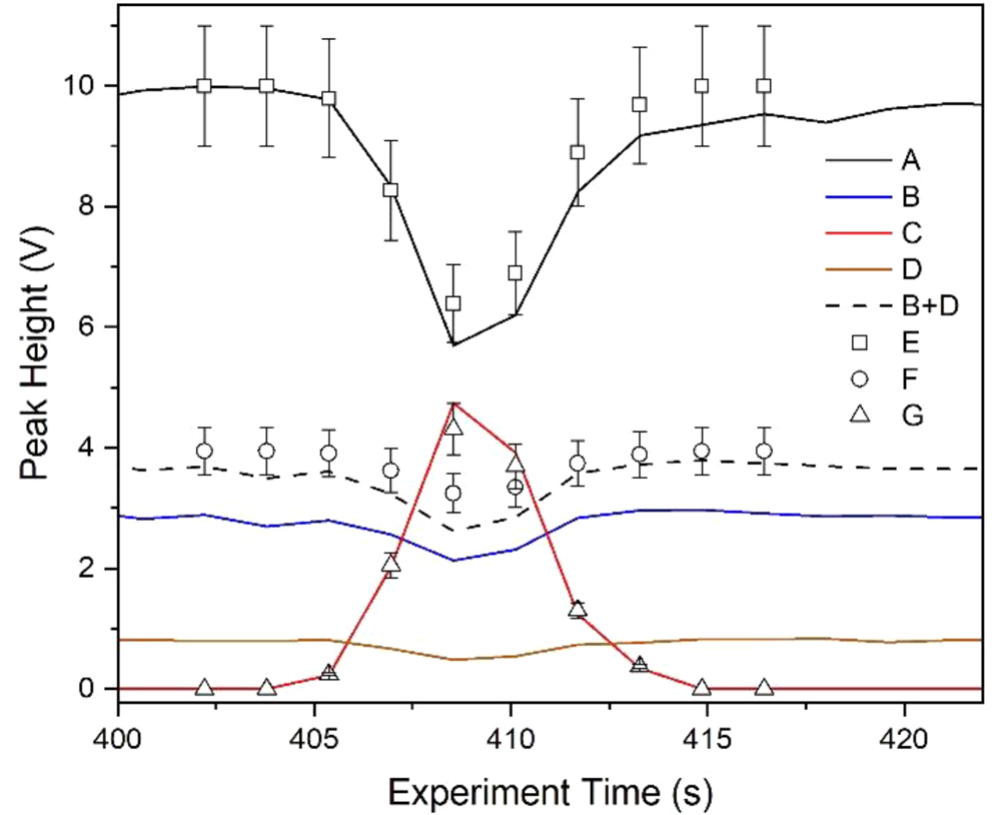
Intensity of mobility peaks with elution time for the binary mixture experiment. Solid lines represent experimental findings with (A) hydrated protons; (B) 6-methyl-5-hepten-2-one; (C) 2,6 di*tert*-butyl-pyridine; (D) 2,7-octanedione contaminant from ketone sample. Open symbols (E–G): computational results normalized to 10 V obtained at 10.5 ms. The dashed line represents the sum of peak heights for the ketone and the contaminant.

In the binary mixture case (blue), the protonated monomer of the amine is seen at 20.14 ms and this is separated at baseline from the peak for ketone. Partially resolved peaks of minor abundance can be observed at the right of the ion peaks for ketone and amine and are considered to have negligible impact with abundances < 5% of the total ion yield. The intensity of the amine peak varied throughout the elution profile, and this also affected the abundance of hydrated proton, the protonated ketone, and that of the impurity. The changes in measured peak heights with elution time for the binary mixture experiment are shown in Figure 7 as solid lines. As the amine elutes from the capillary column, the peak for NH^+^(H_2_O)_*y*_ increases (with decreasing peak heights for H^+^(H_2_O)_*n*_, MH^+^(H_2_O)_*x*_, and 2,7-octanedione contaminant) with elution profiles over 8 s.

The drift tube used for these measurements was based on historic designs for IMS analyzers, and the sample was introduced perpendicular to the flow of ions from the ion source with two immediate impacts. Ions from the corona discharge are shaped into an ion beam of roughly 3–4 mm diameter (Figure S7) where the chromatographic effluent enters the reaction region. Additionally, an effluent flow of 5 mL.min^–1^ produces a velocity of 11 m·s^–1^ at the exit the capillary and impacts the opposite surface wall at 0.3 m·s^–1^ (Figure S8) causing vapor streams and uneven vapor concentrations throughout the analyzer.

Residence times in the ionization region for vapor neutrals entering the drift gas flow can be calculated as 1.1 s by considering the linear velocity of drift gas flow and assuming a uniform distribution of vapor neutrals in the drift tube cross-section. This calculation however is disqualified by the model showing vapor streams and heterogeneous flow patterns caused by jets from the capillary effluent. Consequently, residence times were estimated using comparisons of experimental findings with parameters in the model successively approximated until model and solutions matched within error bars of 10% (Figure 7). Effective residence times of vapors in the source were determined as 10.5 ms within uncertainties of actual vapor concentrations. Predicted and measured abundances matched slightly below 90% for the ketone + contaminant (B+D in Figure 7), and errors in matching could be attributed to the lack of rate coefficient and concentration of the impurity. Rate coefficients in the model were based on a 60 °C gas temperature, which matched the measured temperature of the gas venting the drift tube.

The changes in intensity are characteristic of what is known as competitive ionization in APCI; in this model, we refer to such behavior as selectivity of ionization or distribution of protons. As anticipated from rate coefficients and vapor concentrations, 2,6-di-*tert*-butylpyridine in the source volume leads to reduced abundance of all other ions including reagent ions, protonated monomers, and impurities. Such behavior in Figures 6 and 7 is seen with mixtures throughout measurements using ion mobility spectrometry and API mass spectrometry.

The close alignment of the model and experimental findings is a type of validation of the model given the availability of conditional rate coefficients and details of ions and gas flows in the drift tube. Nonetheless, these parameters diverge from the ideal theoretical considerations.

Despite these approximations, the model can be used to explore broadly reaction times and vapor concentrations with binary mixtures with confidence that the model was validated with specific and known conditional rate coefficients. A more complete experiment is provided in the next section over an extended range of reaction times for an interval of [N] (Figure S9)

### General Model for Ion Abundances for Binary Mixtures

**Figure 8:**
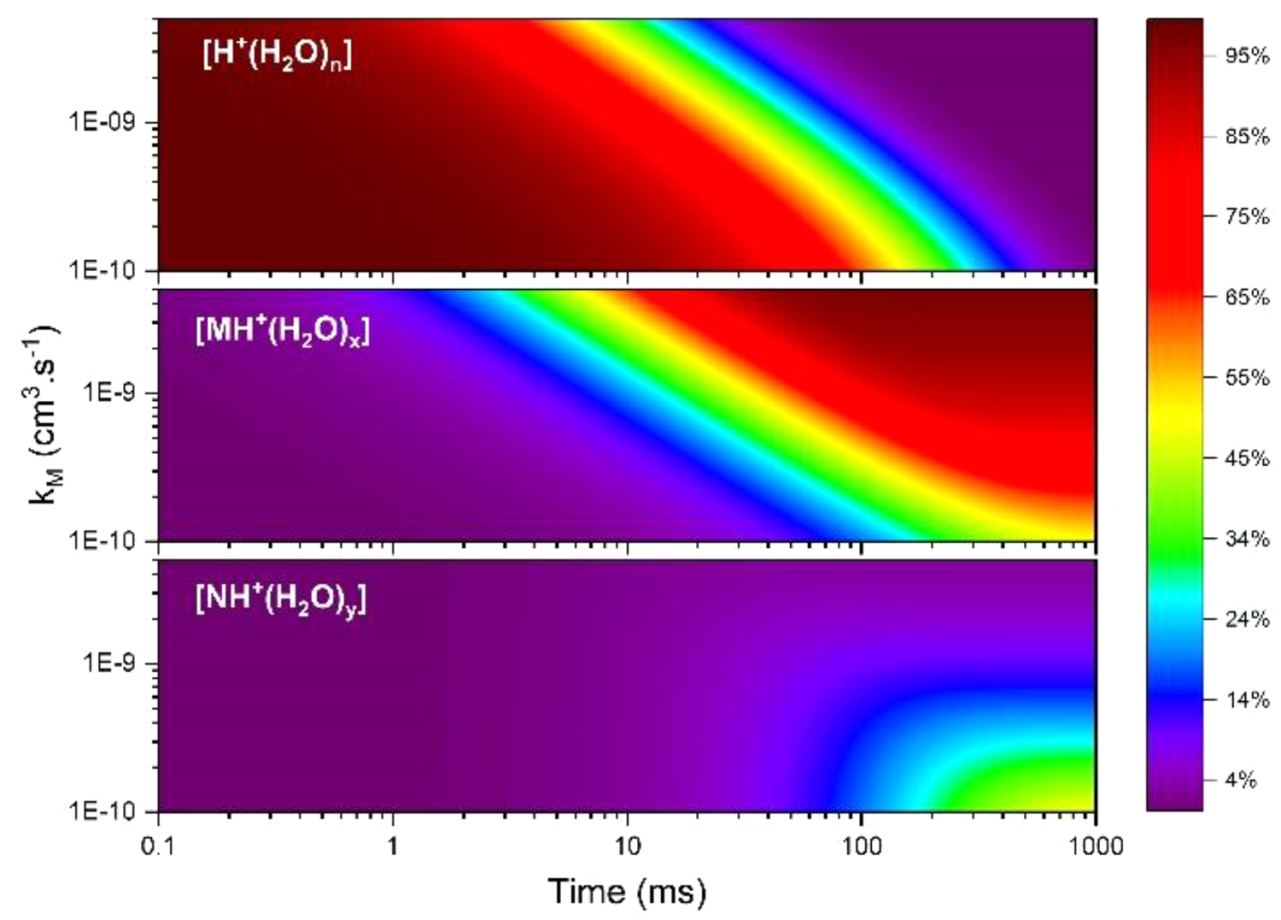
Influence of rate coefficient for M on ion abundances for binary mixture where [M]_initial_ = [N]_initial_ = 1 ppb, [H^+^(H_2_O)_*n*_]_initial_ = 406 ppt, and *k*_N_ fixed at 1 × 10^–10^ cm^3^·s^–1^.

While the findings above were developed for a particular pair of volatile organic compounds, a general form of the model was also developed for a generic binary mixture (M and N), where *k*_N_ = 1 × 10^–10^ cm^3^·s^–1^ and *k*_M_ varied from 1 to 50 × 10^–10^ cm^3^·s^–1^. Vapor concentrations for [M] and [N] were 1 ppb initially, [H^+^(H_2_O)_*n*_] was 1 × 10^10^ cm^–3^ or 406 ppt, and reaction times ranged from 0 to 1000 ms. Cross reactions for 1 ppb equimolar vapor mixture exhibit <5% effect on total product ion abundance at 1000 ms (Figure 5) and were omitted from this model. Ion abundances obtained from this model are listed in Figure 8.

The abundance of hydrated protons inversely matches the appearance of the product ions. The protonated monomer for M first appears at ∼1 ms and saturates at ∼30 ms for maximum *k*_M_. In contrast, when rate coefficients of both substances are equal, *k*_M_ = *k*_N_ = 1 × 10^–10^ cm^3^·s^–1^, [MH^+^(H_2_O)_*x*_] appears first at ∼30 ms, with the same yield as [NH^+^(H_2_O)_*y*_] in this case of an equimolar mixture. Under these conditions, where the total vapor concentration is at 2 ppb, hydrated protons are significantly depleted from 30 to 500 ms, depending on *k*_M_. Product ions for NH^+^(H_2_O)_*y*_ are observed only when *k*_M_ values are lower than 1 × 10^–9^ cm^3^·s^–1^, and reaction times are greater than 30 ms. Since the limiting reagent is hydrated protons, maximum signals for compounds occurs before vapor reactants are totally consumed. The patterns in Figure 8 can be understood through eqs 3 and 4 where product ion formation follows *k*_M_·[M] or *k*_N_·[N] in the absence of cross reactions.^6^ At equal concentrations (=1 ppb), rate coefficients or reactivity of the substance govern the product ion abundances. Rate coefficients of M and N affect the time in which reactant ions are depleted and the relative abundances of formed product ions. Consequently, less reactive constituents in APCI measurements with mixtures may not be measured (or even detected) when rate coefficients differ significantly, even with constituents at equal vapor levels. Although discussed in detail above, the impacts and significance of time on ion abundances and distributions are fully retained in the general model. Reaction times are relatively long at 2 ppb vapor concentration and can be shortened by increasing the density of hydrated protons.

## Conclusions

This model presents the selectivity of ionization introduced by differences in rate coefficients and vapor concentrations, which does not correspond to a simple additive model but rather to a system of differential equations. The close match between the model and experimental findings shows capabilities for quantitative interpretation and prediction of ion abundances with binary mixtures in APCI sources.

This model can be extended to binary mixtures for other VOCs and perhaps adaptable for more complex mixtures providing the availability of experimental “conditional rate coefficients.” Extension to substances and conditions where proton bound dimers are formed is under consideration.

## Supporting Information Available

Design of flows with drift tube for the determination of rate coefficients; schematic of drift tube for ion mobility spectrometry validation of the computational models; ratio of product ions produced in the ambient pressure ion source as a function of time for equimolar vapor concentrations with hydrated protons; spectrum of impurity from GC orbitrap MS analysis; mass spectrum of 6-methyl-5-hepten-2-one headspace sample; flow of hydrated protons from corona discharge from COMSOL modelling in the drift tube of an ion mobility spectrometer; COMSOL models of profile for flows of gas; influence of vapor concentration of N on percent ion yield for protonated monomers of a binary mixture (PDF)

## References

[ref1] Horning E. C.; Horning M. G.; Carroll D. I.; Dzidic I.; Stillwell R. N. New Picogram Detection System Based on a Mass Spectrometer with an External Ionization Source at Atmospheric Pressure. Anal. Chem. 1973, 45 (6), 936–943. 10.1021/ac60328a035.

[ref2] Sunner J.; Nicol G.; Kebarle P. Factors Determining Relative Sensitivity of Analytes in Positive Mode Atmospheric Pressure Ionization Mass Spectrometry. Anal. Chem. 1988, 60 (13), 1300–1307. 10.1021/ac00164a012.

[ref3] Sunner J.; Ikonomou M. G.; Kebarle P. Sensitivity Enhancements Obtained at High Temperatures in Atmospheric Pressure Ionization Mass Spectrometry. Anal. Chem. 1988, 60 (13), 1308–1313. 10.1021/ac00164a013.

[ref4] Covey T. R.; Thomson B. A.; Schneider B. B. Introduction, I. Atmospheric Pressure Ion Sources. Mass Spectrom. Rev. 2009, 28, 870–897. 10.1002/mas.20246. 19626583

[ref5] Rankin-Turner S.; Heaney L. M. Applications of Ambient Ionization Mass Spectrometry in 2020: An Annual Review. Anal. Sci. Adv. 2021, 2, 193–212. 10.1002/ansa.202000135. PMC10989608 38716454

[ref6] Lattouf E.; Anttalainen O.; Kotiaho T.; Hakulinen H.; Vanninen P.; Eiceman G. Parametric Sensitivity in a Generalized Model for Atmospheric Pressure Chemical Ionization Reactions. J. Am. Soc. Mass Spectrom. 2021, 32 (8), 2218–2226. 10.1021/jasms.1c00158. 34264074

[ref7] Brown P.; Watts P.; Märk T. D.; Mayhew C. A. Proton Transfer Reaction Mass Spectrometry Investigations on the Effects of Reduced Electric Field and Reagent Ion Internal Energy on Product Ion Branching Ratios for a Series of Saturated Alcohols. Int. J. Mass Spectrom. 2010, 294 (2–3), 103–111. 10.1016/j.ijms.2010.05.028.

[ref8] Bohme D. K.; Mackay G. I.; Tanner S. D. An Experimental Study of the Gas-Phase Kinetics of Reactions with Hydrated H_3_O^+^ Ions (n = 1–3) at 298 K. J. Am. Chem. Soc. 1979, 101 (14), 3724–3730. 10.1021/ja00508a003.

[ref9] Tanner S. D.; Mackay G. I.; Bohme D. K. A Room-Temperature Study of the Kinetics of Protonation of Formaldehyde. Can. J. Chem. 1979, 57 (18), 2350–2354. 10.1139/v79-378.

[ref10] Mackay G. I.; Tanner S. D.; Hopkinson A. C.; Bohme D. K. Gas-Phase Proton-Transfer Reactions of the Hydronium Ion at 298 K. Can. J. Chem. 1979, 57 (12), 1518–1523. 10.1139/v79-248.

[ref11] Smith D.; Chippendale T. W. E.; Španěl P. Reactions of the Selected Ion Flow Tube Mass Spectrometry Reagent Ions H_3_O^+^ and NO^+^ with a Series of Volatile Aldehydes of Biogenic Significance. Rapid Commun. Mass Spectrom. 2014, 28 (17), 1917–1928. 10.1002/rcm.6977. 25088135

[ref12] Diskin A. M.; Wang T.; Smith D.; Špaň P. A Selected Ion Flow Tube (SIFT), Study of the Reactions of H_3_O^+^, NO^+^ and O_2_^+^ Ions with a Series of Alkenes ; in Support of SIFT-MS. Int. J. Mass Spectrom. 2002, 218, 87–101. 10.1016/S1387-3806(02)00662-0.

[ref13] Zhao J.; Zhang R. Proton Transfer Reaction Rate Constants between Hydronium Ion (H_3_O^+^) and Volatile Organic Compounds. Atmos. Environ. 2004, 38 (14), 2177–2185. 10.1016/j.atmosenv.2004.01.019.

[ref14] Heptner A.; Cochems P.; Langejuergen J.; Gunzer F.; Zimmermann S. Investigation of Ion-Ion-Recombination at Atmospheric Pressure with a Pulsed Electron Gun. Analyst 2012, 137 (21), 5105–5112. 10.1039/c2an35849b. 22977880

[ref15] Valadbeigi Y.; Farrokhpour H.; Rouholahnejad F.; Tabrizchi M. Experimental and Theoretical Study of the Kinetic of Proton Transfer Reaction by Ion Mobility Spectrometry. Int. J. Mass Spectrom. 2014, 369, 105–111. 10.1016/j.ijms.2014.04.011.

[ref16] Ketkar S. N.; Penn S. M.; Fite W. L. Influence of Coexisting Analytes in Atmospheric Pressure Ionization Mass Spectrometry. Anal. Chem. 1991, 63 (9), 924–925. 10.1021/ac00009a018.

[ref17] Puton J.; Holopainen S. I.; Mäkinen M. A.; Sillanpää M. E. T. Quantitative Response of IMS Detector for Mixtures Containing Two Active Components. Anal. Chem. 2012, 84 (21), 9131–9138. 10.1021/ac3018108. 23067016

[ref18] Klee S.; Derpmann V.; Wißdorf W.; Klopotowski S.; Kersten H.; Brockmann K. J.; Benter T.; Albrecht S.; Bruins A. P.; Dousty F.; Kauppila T. J.; Kostiainen R.; O’Brien R.; Robb D. B.; Syage J. A. Are Clusters Important in Understanding the Mechanisms in Atmospheric Pressure Ionization? Part 1: Reagent Ion Generation and Chemical Control of Ion Populations. J. Am. Soc. Mass Spectrom. 2014, 25 (8), 1310–1321. 10.1007/s13361-014-0891-2. 24850441

[ref19] Furey A.; Moriarty M.; Bane V.; Kinsella B.; Lehane M. Ion Suppression ; A Critical Review on Causes, Evaluation, Prevention and Applications. Talanta 2013, 115, 104–122. 10.1016/j.talanta.2013.03.048. 24054567

[ref20] King R.; Bonfiglio R.; Fernandez-Metzler C.; Miller-Stein C.; Olah T. Mechanistic Investigation of Ionization Suppression in Electrospray Ionization. J. Am. Soc. Mass Spectrom. 2000, 11 (11), 942–950. 10.1016/S1044-0305(00)00163-X. 11073257

[ref21] Enke C. G. A Predictive Model for Matrix and Analyte Effects in Electrospray Ionization of Singly-Charged Ionic Analytes. Anal. Chem. 1997, 69 (23), 4885–4893. 10.1021/ac970095w. 9406535

[ref22] Ketkar S. N.; Penn S. M.; Fite W. L. Real-Time Detection of Parts per Trillion Levels of Chemical Warfare Agents in Ambient Air Using Atmospheric Pressure Ionization Tandem Quadrupole Mass Spectrometry. Anal. Chem. 1991, 63 (5), 457–459. 10.1021/ac00005a014.

[ref23] Eiceman G. A.; Blyth D. A.; Shoff D. B.; Snyder A. P. Screening of Solid Commercial Pharmaceuticals Using Ion Mobility Spectrometry. Anal. Chem. 1990, 62 (14), 1374–1379. 10.1021/ac00213a005. 2382838

[ref24] Ochoa M. L.; Harrington P. B. Detection of Methamphetamine in the Presence of Nicotine Using In Situ Chemical Derivatization and Ion Mobility Spectrometry. Anal. Chem. 2004, 76 (4), 985–991. 10.1021/ac035123r. 14961729

[ref25] Verkouteren J. R.; Staymates J. L. Reliability of Ion Mobility Spectrometry for Qualitative Analysis of Complex, Multicomponent Illicit Drug Samples. Forensic Sci. Int. 2011, 206 (1–3), 190–196. 10.1016/j.forsciint.2010.08.005. 20828951

[ref26] Dam R. J. Analysis of Toxic Vapors by Plasma Chromatography. In Plasma Chromatography; Carr T. W., Ed.; Plenum Press: New York, 1984; pp 177–214. 10.1002/bbpc.19840880933

[ref27] Puton J.; Nousiainen M.; Sillanpää M. Ion Mobility Spectrometers with Doped Gases. Talanta 2008, 76 (5), 978–987. 10.1016/j.talanta.2008.05.031. 18761144

[ref28] Waraksa E.; Perycz U.; Namiesnik J.; Sillanpää M.; Dymerski T.; Wojjtowicz M.; Puton J. Dopants and Gas Modifiers in Ion Mobility Spectrometry. TrAC - Trends Anal. Chem. 2016, 82, 237–249. 10.1016/j.trac.2016.06.009.

[ref29] Robb D. B.; Covey T. R.; Bruins A. P. Atmospheric Pressure Photoionization: An Ionization Method for Liquid Chromatography - Mass Spectrometry. Anal. Chem. 2000, 72 (15), 3653–3659. 10.1021/ac0001636. 10952556

[ref30] Rissanen M. P.; Mikkilä J.; Iyer S.; Hakala J. Multi-Scheme Chemical Ionization Inlet (MION) for Fast Switching of Reagent Ion Chemistry in Atmospheric Pressure Chemical Ionization Mass Spectrometry (CIMS) Applications. Atmos. Meas. Technol. 2019, 12 (12), 6635–6646. 10.5194/amt-12-6635-2019.

[ref31] Eiceman G. A.; Nazarov E. G.; Rodriguez J. E.; Stone J. A. Analysis of a Drift Tube at Ambient Pressure: Models and Precise Measurements in Ion Mobility Spectrometry. Rev. Sci. Instrum. 2001, 72 (9), 3610–3621. 10.1063/1.1392339.

[ref32] Jazan E.; Tabrizchi M. Kinetic Study of Proton-Bound Dimer Formation Using Ion Mobility Spectrometry. Chem. Phys. 2009, 355 (1), 37–42. 10.1016/j.chemphys.2008.11.001.

[ref33] Veasey C. A.; Thomas C. L. P. Fast Quantitative Characterisation of Differential Mobility Responses. Analyst 2004, 129, 198–204. 10.1039/b310760d. 14978520

[ref34] Siegel M. W. Atmospheric Pressure Ionization. In Plasma Chromatography; Carr T. W., Ed.; Plenum Press: New York, 1984; pp 96–113.

[ref35] Hunter E. P. L.; Lias S. G. Evaluate Gas Phase Basicities and Proton Affinity of Molecules.Pdf. J. Phys. Chem. Ref. Data 1998, 27 (3), 413–656. 10.1063/1.556018.

[ref36] Bohme D. K.; Mackay G. I.; Tanner S. D. An Experimental Study of the Gas Phase Kinetics of Reactions with Hydrated H_3_O^+^ Ions (N = 1–3) at 298K. J. Am. Chem. Soc. 1979, 101 (14), 3724–3730. 10.1021/ja00508a003.

[ref37] Bohme D. K.; Mackay G. I.; Schiff H. I. Determination of Proton Affinities from the Kinetics of Proton Transfer Reactions. VII. The Proton Affinities of O_2_, H_2_, Kr, O, N_2_, Xe, CO_2_, CH_4_, N_2_O, and CO. J. Chem. Phys. 1980, 73 (10), 4976 10.1063/1.439975.

[ref38] Safaei Z.; Willy T. J. T. J.; Eiceman G. A.; Stone J. A. A.; Sillanpää M. Quantitative Response in Ion Mobility Spectrometry with Atmospheric Pressure Chemical Ionization in Positive Polarity as a Function of Moisture and Temperature. Anal. Chim. Acta 2019, 1092, 144–150. 10.1016/j.aca.2019.09.040. 31708027

[ref39] Mautner M. The Ionic Hydrogen Bond and Ion Solvation. 1. NH^+^···O, NH^+^···N, and OH^+^···O Bonds. Correlations with Proton Affinity. Deviations Due to Structural Effects. J. Am. Chem. Soc. 1984, 106 (5), 1257–1264. 10.1021/ja00317a015.

[ref40] Ewing R. G.; Eiceman G. A.; Stone J. A. Proton-Bound Cluster Ions in Ion Mobility Spectrometry. Int. J. Mass Spectrom. 1999, 193 (1), 57–68. 10.1016/S1387-3806(99)00141-4. 11543494

