# Supplementary material for: Quantitative Distributions of Product Ions and Reaction Times with a Binary Mixture of VOCs in Ambient Pressure Chemical Ionization
Source: J Am Soc Mass Spectrom. 2023 Jul 15;34(8):1768–77. doi: 10.1021/jasms.3c00189 (PMC10401699; doi:10.1021/jasms.3c00189)
Supplement: Supplementary file 1 — js3c00189_si_001.pdf [file js3c00189_si_001.pdf]

Supporting information for

Quantitative Distributions of Product Ions and Reaction Times with a Binary Mixture of VOCs in Ambient Pressure Chemical Ionization

Elie Lattouf*, [1], Osmo Anttalainen[1], Oliver Hecht[2], Bert Ungethüm[2], Tapio Kotiaho[3], Hanna Hakulinen[1], Paula Vanninen[1] and Gary Eiceman[1,4]

[1)] VERIFIN, Finnish Institute for Verification of the Chemical Weapons Convention, Department of Chemistry, University of Helsinki, FI-00014 Helsinki, Finland

[2)] AIRSENSE Analytics Gmbh, Hagenower Straße 73, 19061 Schwerin, Germany

[3)] Drug Research Program and Division of Pharmaceutical Chemistry and Technology and Department of Chemistry, University of Helsinki, FI-00014 Helsinki, Finland

[4)] New Mexico State University, 1175 N Horseshoe Dr., Las Cruces, NM 88003,USA

* Author to whom correspondence should be addressed: elie.lattouf@helsinki.fi

Table of Contents

Figure S1 2

Figure S2 2

Figure S3 3

Figure S4 3

Figure S5: 4

Figure S6: 4

Figure S7 5

Figure S8 6

Figure S9 7

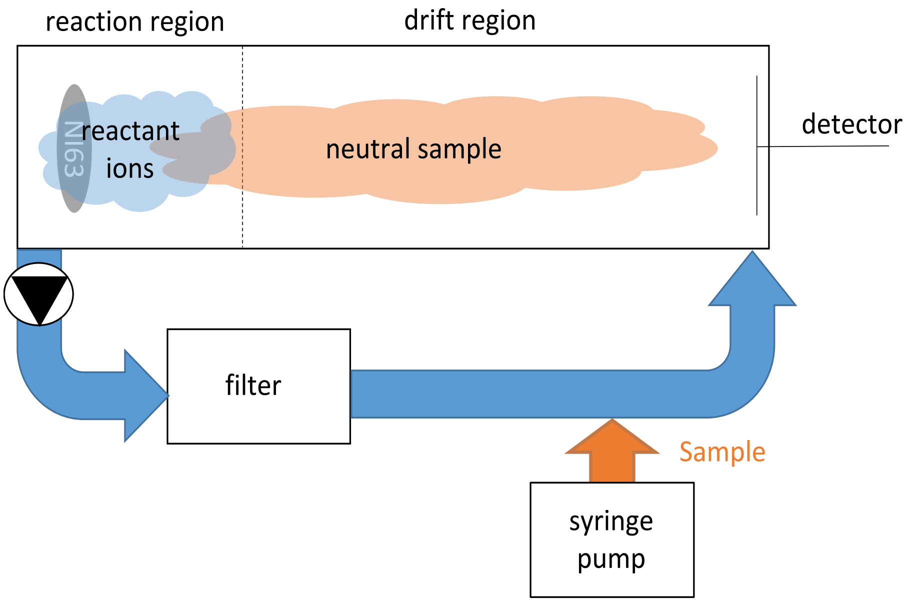

Figure S1: Design of flows with drift tube for the determination of rate coefficients for individual substances using an ion mobility spectrometer at ambient pressure.

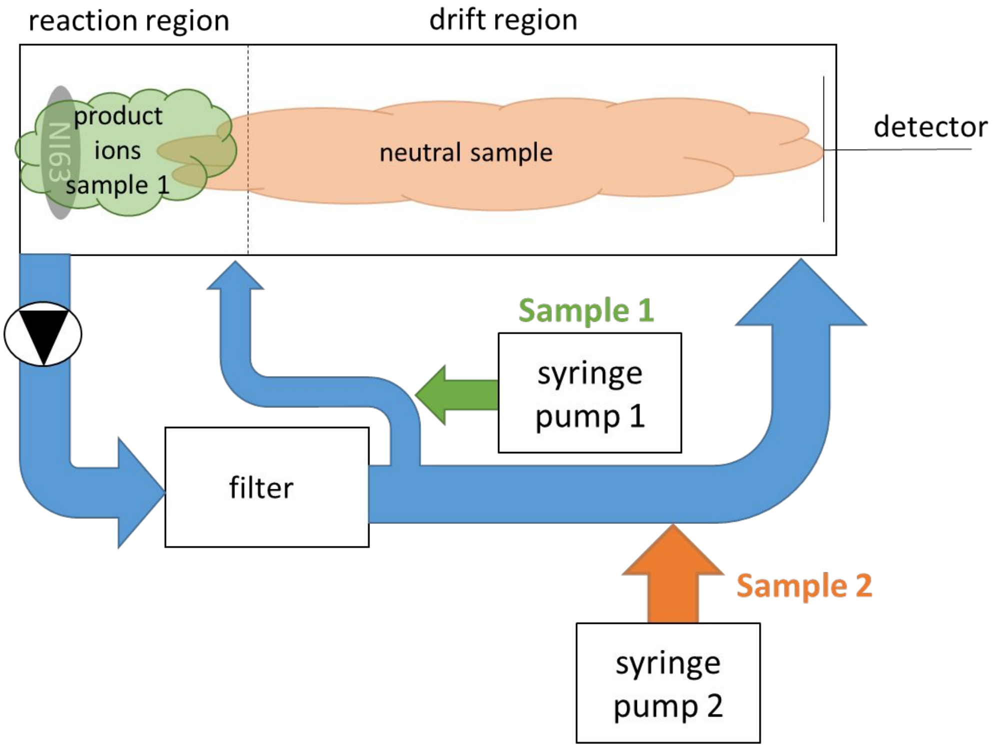

Figure S2: Design of flows with drift tube for the determination of rate coefficients for cross reaction (Equation 5) using an ion mobility spectrometer at ambient pressure. Vapours of 6-methyl-5-hepten-2-one were added into the ion source region with syringe pump 1 and vapours of 2,6-di-*tert*-butyl-pyridine were added using syringe pump 2 into the drift gas flows. Flows vented from the drift tube were recirculated after purification in a molecular sieve filter.

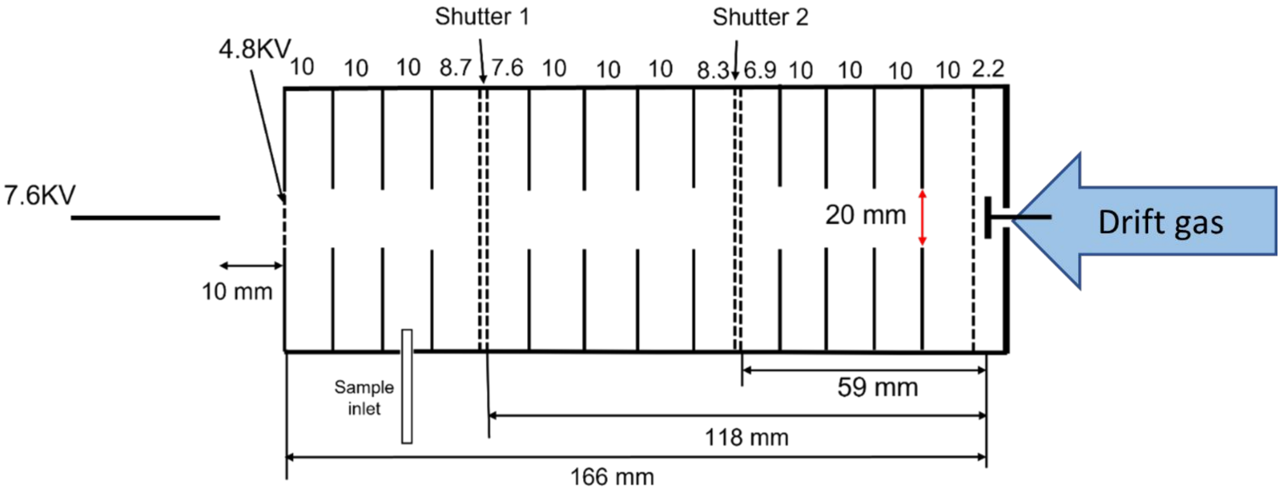

Figure S3: Schematic of drift tube for ion mobility spectrometry validation of the computational models. Drift gas was purified air with unidirectional flow as shown. A flow of ketone vapour in air was continuously introduced through the sample inlet which also received effluent from the gas chromatograph containing amine eluted from a capillary column.

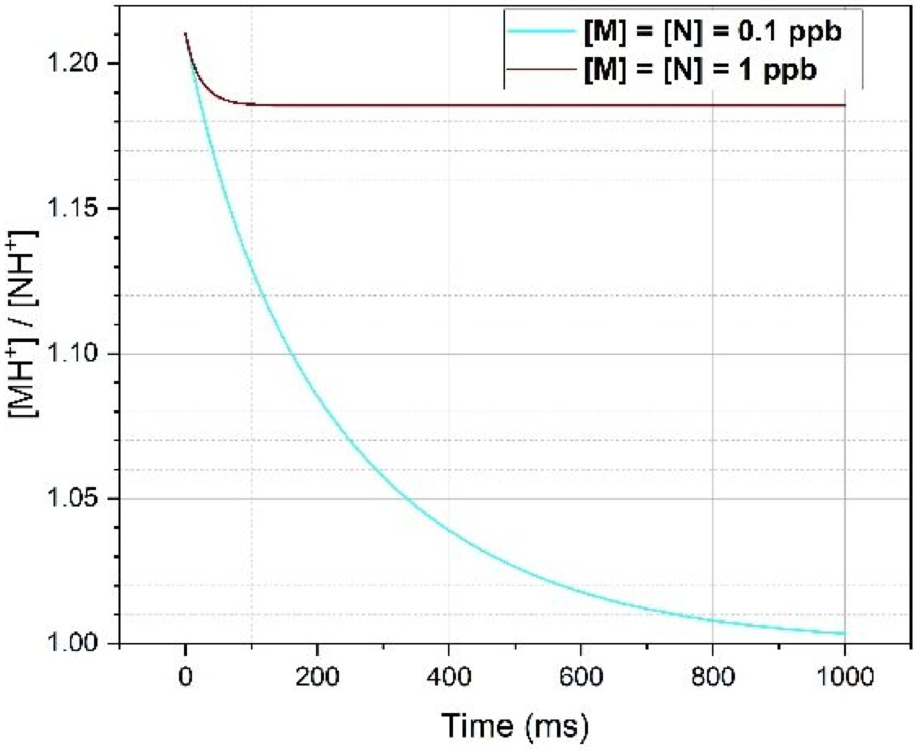

Figure S4: Ratio of product ions produced in the ambient pressure ion source as a function of time for equimolar vapour concentrations with hydrated protons in excess (light blue) and hydrate protons as limiting reagent (brown).

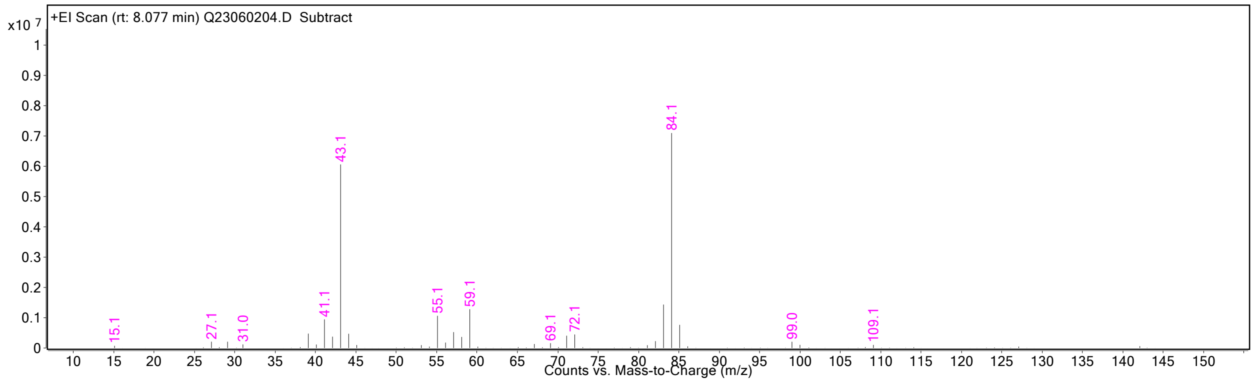

Figure S5: Spectrum of impurity from GC orbitrap MS analysis with electron impact ionization of 6-methyl-5-hepten-2-one headspace sample. Impurity was determined as 2,7 octanedione (https://pubchem.ncbi.nlm.nih.gov/compound/2_7-Octanedione#section=GC-MS). See Figure S6.

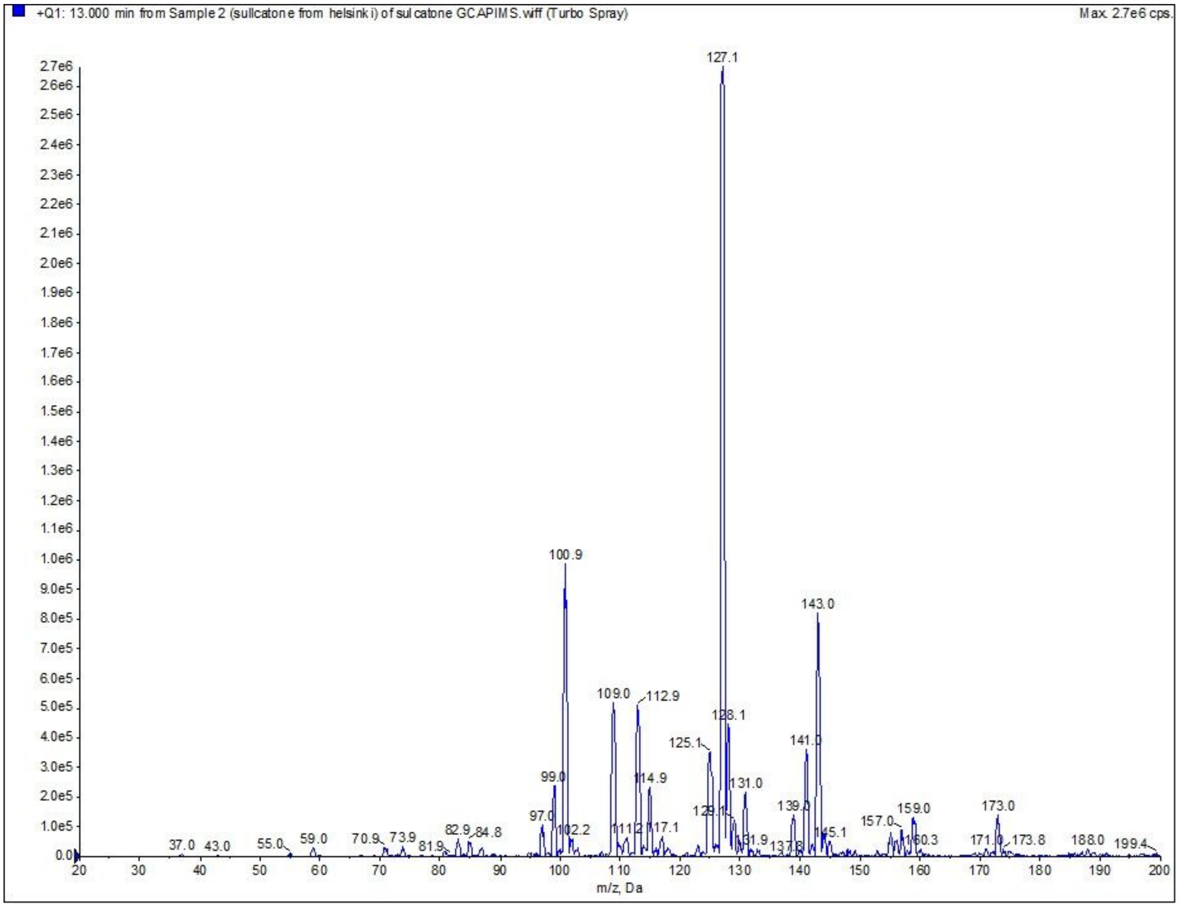

Figure S6: Mass Spectrum of 6-methyl-5-hepten-2-one headspace sample showing the protonated monomer at 143 Da for the 2,7 octanedione impurity.

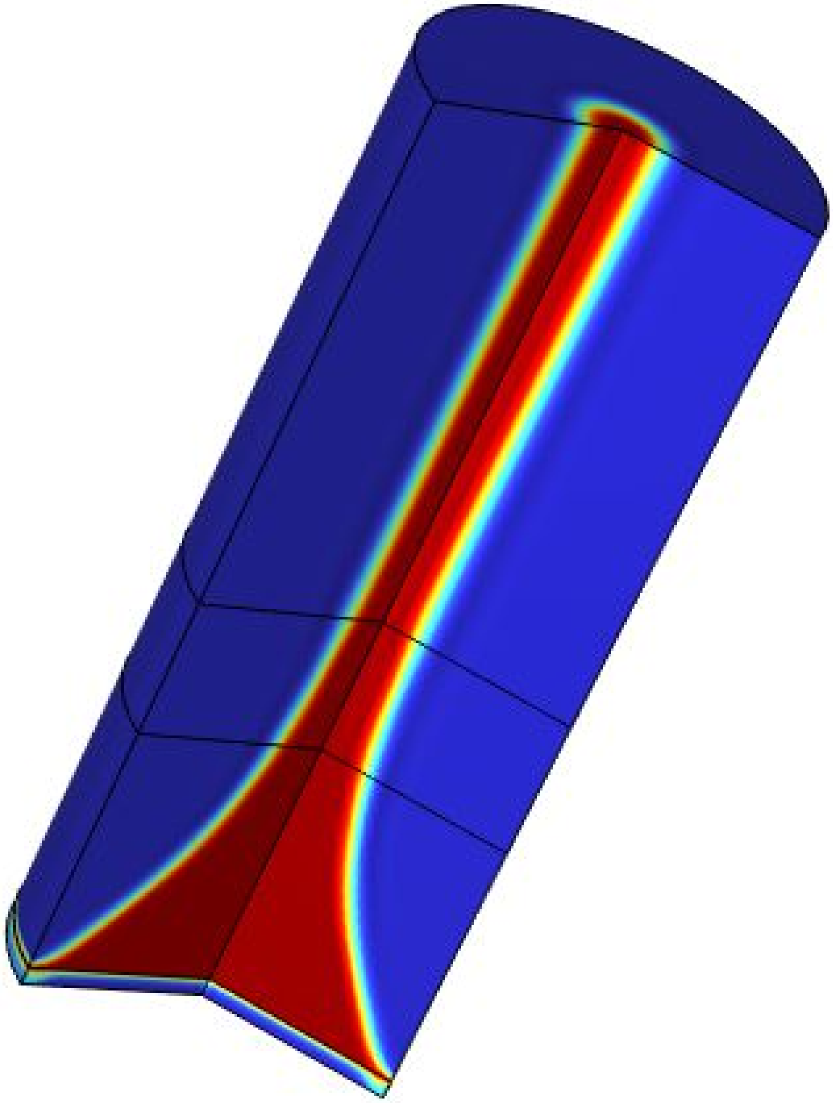

Figure S7: Flow of hydrated protons from corona discharge from COMSOL modelling in the drift tube of an ion mobility spectrometer. The ions form a beam in the centre of the drift tube. Ion density colour coded with red highest density and blue lowest density.

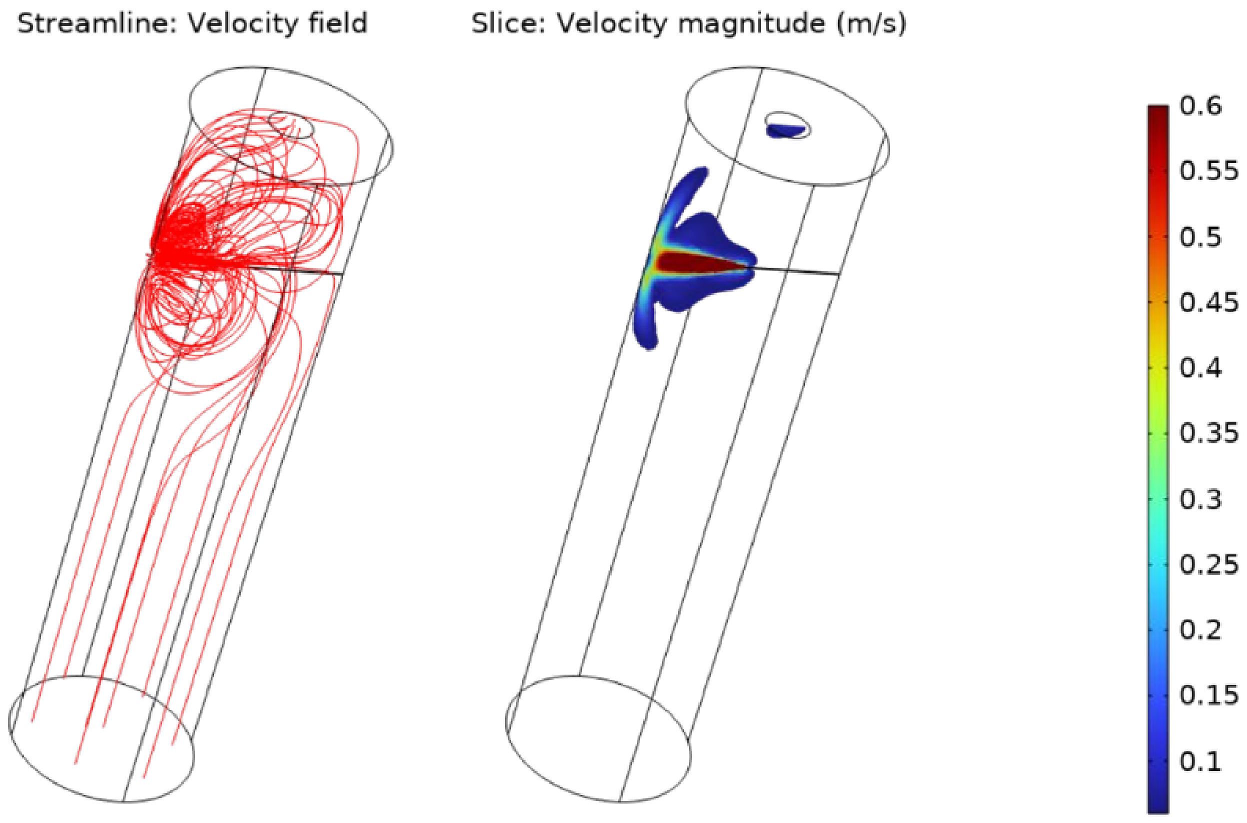

Figure S8: COMSOL models of profile for flows of gas showing jets of neutrals from effluent of capillary column entering the drift tube, impacting the inner wall of the drift tube, and spreading into the inner volume of the reaction region.

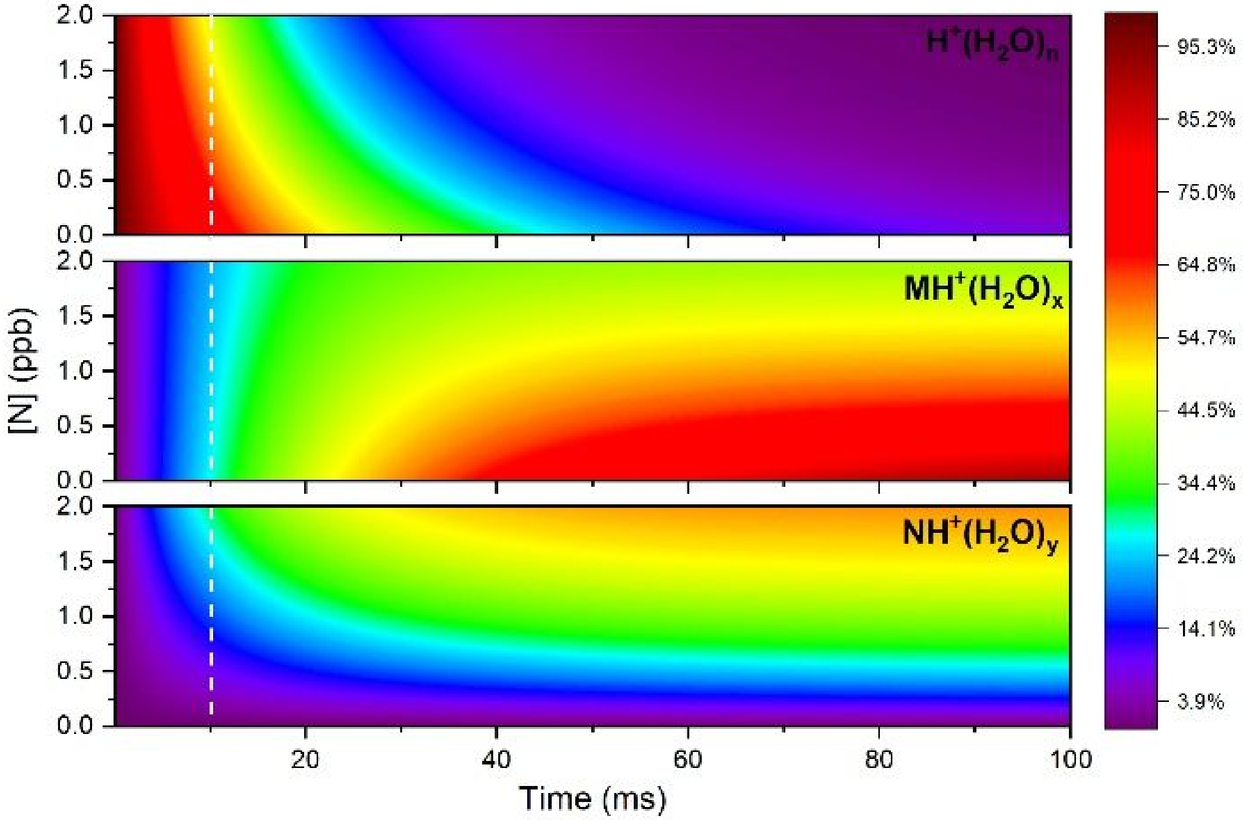

Figure S9: Influence of vapour concentration of N on percent ion yield for protonated monomers of binary mixture. [M] = 1.22 ppb and [N] = 0 - 2 ppb, matching experimental settings. The vertical dashed line at t = 10.5 ms corresponds to calculated ion yields in Figure 7.
